# Barriers to weight loss among community health center patients: qualitative insights from primary care providers

**DOI:** 10.1186/s40608-016-0123-3

**Published:** 2016-10-21

**Authors:** Rebecca C. Woodruff, Gillian L. Schauer, Ann R. Addison, Ajay Gehlot, Michelle C. Kegler

**Affiliations:** 1 Department of Behavioral Sciences and Health Education, Rollins School of Public Health, Emory University, 1518 Clifton Rd NE, GCR 569, Atlanta, GA 30322 USA; 2 Department of Health Services, School of Public Health, University of Washington, 1959 NE Pacific St, Magnuson Health Sciences Center, Rm H-680, Box 357660, Seattle, WA 98795-7660 USA; 3 Primary Care of Southwest Georgia, Inc., 360 College St., Blakely, GA 39823 USA; 4 Southwest Georgia Health Care, 804 E 16th Ave, Cordele, GA 31015 USA

**Keywords:** United States, Community health centers, Primary health care, Qualitative research, Body weight, Weight loss

## Abstract

**Background:**

Community Health Centers (CHCs) are important settings for obesity prevention and control. However, few studies have explored the barriers that CHC clinicians perceive their patients face in maintaining a healthy weight.

**Methods:**

Semi-structured in-depth interviews were conducted with thirty physicians, physician assistants, and nurse practitioners recruited from four Community Health Centers (CHCs), located in a rural, southwestern region of the state of Georgia, US. Interviews were digitally recorded, transcribed verbatim, and thematically analyzed.

**Results:**

Clinicians perceived that their patients face numerous individual, interpersonal, and community-level barriers to weight loss. Perceived individual-level barriers included interrelated aspects of poverty and limited motivation to lose weight. Perceived interpersonal barriers included social and cultural norms, such as positive associations with larger body sizes, negative associations with smaller body sizes, lack of awareness of obesity as a problem, and beliefs regarding hereditary or generational body types. Perceived community-level barriers included limited healthy food options and aspects of the local food culture in the Southern US.

**Conclusions:**

Clinicians perceived that their patients face barriers to weight loss at multiple levels of the social ecology, including individual, social, and environmental factors. Results may partly explain limited provision of weight counseling in CHCs and suggest opportunities for intervention.

## Background

According to recent national estimates, over one-third of adults in the United States (US) are obese, which puts them at elevated risk for developing many chronic diseases, including type II diabetes, cardiovascular disease, and some forms of cancer [1, 2]. By 2030, 65 million American adults are estimated to be obese, with attributable medical spending projected to increase by $48–66 billion annually [3]. Low-income, racial and ethnicity minority, and rural populations bear a disproportionate burden of the obesity epidemic in the US [4, 5].

Community Health Centers (CHCs), also known as Federally Qualified Health Centers, are an important setting for obesity prevention and control in the US. Established in 1965, CHCs are a network of safety net clinics that are administered by the Health Resources and Services Administration (HRSA) to provide primary and preventive health care services to uninsured and medically underserved populations [6]. HRSA estimates that in 2014, 22.9 million Americans received medical care at approximately 1300 CHCs nationwide [7]. According to HRSA, 27.9 % of CHC patients are uninsured, 71.2 % are at or below 100 % of the federal poverty level, and 62.2 % are from racial or ethnic minority populations [7]. Approximately 48.0 % of CHC patients are estimated to be obese, suggesting that CHCs are an important setting to address and treat obesity [8–10]. Given that the demand for medical services provided by CHCs is expected to grow now that millions of Americans are newly insured under the Patient Protection and Affordable Care Act [6], a greater understanding of obesity control efforts at CHCs has emerged as an important area of research.

Weight counseling is the traditional approach for addressing obesity in clinical settings. The United States Preventive Services Task Force recommends that clinicians screen all patients for obesity and offer intensive counseling, which has been shown to result in modest weight loss over time [11]. Additionally, HRSA now requires that CHCs report the proportion of patients with an elevated Body Mass Index (BMI) who have a documented follow-up plan as a clinical and financial performance measure, indicating an increased emphasis on diagnosing obesity and providing appropriate follow-up care in this setting [10, 12, 13]. However, obesity diagnoses and the provision of weight counseling remains low at clinics nationwide, including at CHCs [7, 14–16]. According to clinical service data from HRSA, in 2014, 56.1 % of adult CHC patients received weight screening and follow-up [7]. According to surveys with overweight and obese CHC patients, 45.2 % reported that a physician had told them that they were overweight [16]. These findings have led researchers to explore the barriers that prevent clinicians from providing weight counseling during clinical visits.

Prior research has documented that clinicians perceive many barriers to providing weight counseling during primary care visits, including lack of time, competing clinical priorities other than obesity, insufficient reimbursement, and lack of community-based resources to which they can refer patients [12, 17–22]. Several studies have also found that clinicians perceive complex social, cultural, economic, and environmental determinants of obesity [12, 17–19], though no studies have attempted to characterize clinicians’ perceptions of these determinants in depth. A greater understanding of the barriers that CHC clinicians perceive their patient populations face in maintaining a healthy weight may provide additional insight into reasons why weight counseling remains low at CHCs and suggest opportunities for intervention. The purpose of this study was to use qualitative research methods to describe in depth the barriers that CHC clinicians perceive their patient populations face in maintaining a healthy weight.

## Methods

### Participants and recruitment

In fall 2012, participants were recruited via email from 30 individual clinics that were part of four multi-clinic CHCs located in the predominately rural, southwestern region of the state of Georgia. Low-income African Americans were the majority of the patient population served by these clinics. Eligible participants were physicians, physician assistants, and nurse practitioners who spoke English and served primarily adult patient populations. Interviews conducted with clinicians who serve pediatric patients were excluded from this analysis due to thematic differences in barriers that pediatric patients face in maintaining a healthy weight.

### Data collection

A total of 30 clinicians completed semi-structured in-depth interviews (mean duration 34 min, range 25–48 min) and a brief questionnaire about demographic characteristics either in person or by telephone. Participants who completed the interview in person provided written informed consent, and participants who completed the interview by telephone provided verbal informed consent prior to completing the interview. All participants received a $25 gift card as an incentive for participating in this study. The interview guide included open-ended questions about when and to whom clinicians provide weight counseling, how clinicians counsel on weight, treatment and referral resources, and barriers to providing weight counseling. The interview guide was developed in consultation with representatives from the participating clinics (e.g., clinic CEOs, clinical directors, project managers). This analysis reports results regarding the barriers that CHC clinicians perceive their patients face in maintaining a healthy weight. Results regarding when, to whom, and how clinicians provide weight counseling has been reported in a prior publication [22]. Barriers to providing weight counseling were assessed using this question: “In general, what is the biggest challenge you face in bringing up or addressing weight with your patients?” All study procedures were approved by the Emory University Institutional Review Board (Study ID: 00059222).

### Data analysis

All interviews were digitally recorded, transcribed verbatim, coded using qualitative software, and thematically analyzed. The codebook was developed iteratively by first creating a set of a priori codes based on the interview guide and then identifying inductive codes based on emerging qualitative themes. Inductive codes were identified by reading a selection of transcripts, and were refined using a double-coding process. Two investigators (GLS and RCW) each coded six transcripts and met to resolve any coding discrepancies and revise the codebook. Once the codebook was finalized, one investigator (GLS) coded all transcripts and another investigator (RCW) double-coded one-third of the transcripts for reliability [23]. One investigator (RCW) queried the final dataset to retrieve code reports, read the code reports, and wrote memos about each of them. Themes that emerged from this process were confirmed with a second investigator (GLS), and were categorized according to the levels of the Social Ecological Framework [24]. All themes discussed in this manuscript were reported by at least five respondents, and strong themes were reported by at least half of the sample.

Although clinicians in this study reported a range of barriers to providing weight counseling, this paper specifically focuses on the barriers they perceive their patients face in losing weight. We chose to focus on this subset of barriers, as we identified this as an important gap in the existing research literature. We used MaxQDA software (Version 10, 2013, VERBI Software, Berlin, Germany) for qualitative analysis and SAS 9.3 (SAS Institute, Cary, NC) to analyze demographic variables.

## Results

### Demographics

Participating clinicians were physicians (*n* = 14, 46.7 %), physician assistants (*n* = 11, 36.7 %), or nurse practitioners (*n* = 5, 16.7 %) who provided care for both children and adults (*n* = 18, 60.0 %) or to adults only (*n* = 12, 40.0 %). Clinicians reported that they had been practicing medicine for between 1 and 37 years, with a mean length of 13.3 years (standard deviation = 10.9). The sample included both male (*n* = 14, 46.7 %) and female (*n* = 16, 53.3 %) clinicians. Most clinicians were ages 36–45 (*n* = 13, 43.3 %). Eight clinicians were age 56–65 (26.7 %), six were age 18–35 (20.0 %), and three were age 46–55 (10.0%). The majority of participants reported their race as non-Hispanic white (*n* = 20, 66.7 %), followed by non-Hispanic Black (*n* = 5, 16.7 %), non-Hispanic Asian (*n* = 3, 10.0 %), and Hispanic (*n* = 2, 6.7 %).

Clinicians identified individual, interpersonal, and community-level barriers that they perceive their patient populations face in maintaining a healthy weight (Table 1).

**Table 1:** Barriers to healthy weight maintenance that primary care providers practicing at community health centers perceive their patient populations face

Level of the Social Ecology	Identified barrier
Individual	• Limited economic resources to access weight-loss refferals and healthy food options.
	• Limited educational attainment and literacy levels.
	• Lack of interest and motivation to lose weight and comply with clinical advice regarding weight loss.
Interpersonal	• Positive associations with larger body sizes and negative associations with smaller body sizes.
	• Lack of awareness of overweight and obesity because of the high prevalence of these conditions within patient communities.
	• Beliefs about hereditary or generational body types.
Community	• Limited availability of healthy foods.
	• Southern food culture.

### Individual-level barriers

Clinicians identified a number of individual-level barriers that they perceive their patients face in maintaining a healthy weight, including multiple intersecting aspects of poverty, such as limited economic resources, education, and literacy and lack of motivation to lose weight and to adhere to weight loss counseling recommendations.

#### Limited economic resources

Patients’ limited economic resources emerged as a strong theme within the individual-level barriers reported by clinicians. For example, clinicians perceived that patients’ scarce economic resources profoundly limited their ability to access health-promoting resources and services within their communities, such as medical appointments, visits with nutritionists and dieticians, and gym memberships. One physician assistant summarized this by saying, “If your patient can’t get that [weight loss referral] service because of a financial reason, it’s the same as [if] it didn’t exist.” Clinicians also reported that healthy food options cost more than the ubiquitous, less expensive processed food items available within patients’ communities, and that these cost disparities prevented patients from acting on clinical advice regarding dietary modification. In the words of one nurse practitioner, “I mean, [try telling] somebody to go, eat fresh fruits and vegetables when you can buy five boxes of macaroni and cheese for a dime a box, you know? Money is a huge factor.”

#### Limited education and literacy

Limited educational attainment and literacy levels also emerged as a strong individual-level theme. Clinicians described that providing nutrition-related health education is often complex and time-consuming, that time to provide such counseling during clinical encounters is limited, and that low literacy further complicates their ability to deliver counseling effectively. As one nurse practitioner put it, “If you’ve got 15–20 patients on the schedule and you’re trying to do in-depth teaching to somebody with a 9^th^ grade education who’s just not getting it, it’s hard.” Clinicians also reported that technical nutrition concepts commonly used in health education, such as serving sizes, calorie counting, macronutrient intake, and diabetic exchanges are complex to explain and do not necessarily provide patients with the information that they need to make changes to their diets. One physician illustrated this challenge by reporting that, “Patients just want to know what to eat… something simple that’s at an 8^th^ grade level. ‘Eat this. Don’t eat this.’ They don’t need to know about portions and serving sizes and exchanges.” Another clinician suggested that providing nutrition education can be challenging when health care providers also struggle to understand the nutrition concepts they are supposed to teach patients: “We have some simple [resources], like the plate diagram… And that’s pretty simple and easy to understand, but anything that talks about calories and servings sizes… it’s a huge challenge. Even in the medical field we’re not great with what a serving size is, so how can we expect [our patients] to be?”

Clinicians offered specific recommendations for health education materials that would better aid them in counseling low-literacy patient populations about weight loss. Suggestions included materials that rely less on text and more on graphics (e.g., diagrams of healthy plates), focus on specific behavior change recommendations (e.g., remove skin from chicken before cooking, replace sugar with artificial sweeteners when making sweet tea), simple advice for healthier food choices (e.g., choose baked or broiled chicken over fried chicken), and lists of foods to eat more of or to avoid. Some, but not all, clinicians reported that they had already begun developing such materials themselves: “You really need to simplify it all. That’s why I did a list of food that they need to try to eat more, and the things that [they] really need to avoid, because otherwise it will be really complicated. I mean, if you say, ‘Oh you need to count calories’ or ‘You need to count carbs,’ they won’t do it.”

#### Lack of motivation

Lack of motivation to lose weight also emerged as an individual-level theme. Clinicians reported that in general, they perceived their patients to lack interest in weight loss and motivation to comply with clinical advice to lose weight, such as making changes to their diets. In the words of one physician, “If the patient is not interested, you can’t do anything about it. You can talk to them until you’re blue in the face, but it’s not going to happen if they’re not interested.” According to several clinicians, a diagnosis of a chronic disease, such as pre-diabetes or diabetes often increased patient interest in losing weight. As one physician put it, “Sometimes [preventing diabetes is] the biggest factor that they’d be interested in. You know, ‘I don’t want to be a diabetic! I don’t want to stick myself with needles!’” However, clinicians reported that this motivation is often short-lived and even very motivated patients often have great difficulty sustaining behavior change over longer periods of time.

Clinicians also perceived that lack of patient motivation and compliance with clinical advice were not unique to weight loss counseling, but were also problems in other aspects of disease management, including medication adherence and appointment keeping. Even so, some clinicians experienced these barriers as deeply demoralizing. In the words of one nurse practitioner, “It gets discouraging for me… I tell them they need to lose, lose, lose, lose, and it kind of makes me feel almost like a failure that I’m not getting through to my patient that they need to lose weight… It makes me not want to bring it up as much. I still do it, but in the back of my mind I’m talking to deaf ears, and they’re not going to do what I tell them to do. I get real pessimistic about it.”

### Interpersonal-level barriers

Clinicians identified several interpersonal influences that they believe prevent their patients from maintaining a healthy weight, including social norms regarding body size and those that arise from the high prevalence of obesity within patients’ communities.

#### Social norms regarding weight

Social norms emerged as a strong interpersonal-level theme. Clinicians typically talked about normative influences in two distinct ways. The first of these ways focused on the meaning of various body sizes within patients’ communities. For example, clinicians reported that many patients associate body sizes that fall into the clinically overweight or obese BMI categories with a variety of positive attributes, including health, prosperity, affluence, and power. Conversely, clinicians reported that many patients associate body sizes that fall within the clinically normal BMI category with negative attributes, such as sickness, weakness, or a scrawny or “skin and bones” appearance. One physician described these social influences by saying, “Unfortunately, the culture that we live in is that a skinny baby is a sick baby and the more chubby a baby is, the more healthy the baby looks. And that starts from the cradle and it goes into our older folks - they still have the concept that you need to have some meat on your bones.” Some, though not all clinicians perceived these norms to exist among specific racial or ethnic groups and suggested that these normative beliefs may have deep cultural roots.

Clinicians also spoke about social norms in terms of patients’ weight-related expectations that result from the high prevalence of overweight and obesity in their communities. For example, clinicians reported that some patients may simply not know that they are overweight or obese because these conditions are so common in their communities. As one physician put it, “The perception of obesity just doesn’t exist where we are.” Some, but not all clinicians noted that these normative influences were particularly powerful when patients' family members tended to be overweight or obese, creating a feeling of a hereditary or generational body type. According to one physician, “I think one of the biggest barriers are patients’ own preconceived notions about weight. You know, [the preconceived notion that] I can’t lose weight [because] I’ve been fat all my life. My mother’s fat, my father’s fat, [so] I must be destined to be fat.” These social norms interfered with clinicians’ weight counseling efforts, as weight loss and behavior change recommendations often conflicted with patients’ perceptions of their own weight and with messages that patients receive from other important social referents within their families, social networks, and communities. One physician illustrated this tension by saying, “We do try to tell [patients] they are overweight, but everyone else tells them they’re ok or might be underweight.”

### Community-level barriers

Clinicians identified two main community-level barriers that they believe their patients face: the limited availability of healthy food options within patients’ communities and aspects of Southern food culture.

#### Limited availability of healthy food options

Unsupportive food environments attributable to a limited availability of healthy foods emerged as a community-level theme. Clinicians pointed out that while fast food restaurants and smaller grocery stores that tend to have a limited supply of healthy options are widely available, few supermarkets, farmers’ markets, and restaurants that sell a greater variety of healthy foods exist in the communities they serve. As one clinician put it, “We don’t have a lot of the great, like farmers’ markets and groceries. We just have little grocery stores that don’t have a whole lot of selection. You find yourself having to go to [the two nearest cities] just to get half the healthy stuff you really should eat.” The lack of healthy food options, combined with the high cost of healthy food, prevented many clinicians from effectively counseling their patients about how to modify their diets to support weight loss.

Clinicians also identified aspects of Southern food culture as a theme related to community-level barriers to healthy eating and weight maintenance. One physician assistant described this barrier by saying, “It’s first of all just the food culture in Georgia. A lot of people, they eat a lot of fast food. Their ways of cooking things are a lot of fried foods, fried pork ribs, fried pork chops, all that stuff. Very little fruits, vegetables, and whole grains. Lots of sugar sweetened beverages. A diet that’s predominately meat-based.” Some clinicians found it difficult to counsel patients to stop eating their favorite foods because, in the words of one physician, “For a lot of [patients], it’s just one of the few pleasures they’ve got.” Others chose to counsel patients on ways to make their favorite foods healthier. As one physician put it, “[My patients] don’t want to give up their sweet tea because they love it. It’s the South. It’s sweet tea. So you just say, instead of [sugar], put [an artificial sweetener] or something else and you’re just reducing the calories.”

## Discussion

To our knowledge, this study is the first in-depth exploration of clinicians’ perspectives on the barriers that they perceive their patients face in losing weight. An important finding from this study is that CHC clinicians perceive that their patients face barriers at multiple levels of the social ecology, including at the individual, interpersonal, and community levels [24]. At the individual level, CHC clinicians perceived that patients’ limited economic resources, limited literacy and educational attainment, and lack of motivation acted as barriers to maintaining a healthy weight. At the interpersonal and community levels, CHC clinicians perceived non-health-promoting social norms and characteristics of community food environments to be important barriers their patients face. These results confirm findings from prior qualitative studies that clinicians perceive complex social, cultural, economic and environmental determinants of obesity [12, 17–19], and extend this previous body of work by characterizing these barriers in greater depth.

Regarding individual-level barriers, clinicians perceived that limited economic resources prevented their patients from acting on weight counseling advice and accessing health-promoting resources and services within their communities. This is an important finding given that many recommendations that are provided to patients during weight counseling involve some cost. For example, clinicians often counsel patients to include healthier foods, such as fruits and vegetables, in their diets but noted that these food items are expensive relative to other less healthy food options available in patients’ communities. Additionally, clinicians often encourage patients to schedule follow-up medical visits for obesity counseling or to meet with nutritionists or dieticians, but these visits often require co-pays or out-of-pocket payments that patients may be unable to afford. These results suggest that clinicians may be less likely to provide obesity counseling to patients that they believe do not have the financial resources needed to act on their advice. This finding suggests that clinicians may benefit from the development of counseling materials and recommendations that include cost-neutral actions that patients can take to help them achieve a healthy weight.

Another important finding from this study is that clinicians perceived limited literacy and education as barriers that their patients face in maintaining a healthy weight. Clinicians acknowledged that they have print materials to assist them in providing nutrition counseling to patients, but noted that these materials are complex and time-consuming to explain to patients, particularly to those with limited literacy levels and educational attainment. Additionally, clinicians pointed out that these materials may not provide the information patients need to make health-promoting changes to their diets. As an alternative to the traditional nutrition education materials focused on serving sizes, calorie counting, macronutrient intake, and diabetic exchanges, participants in this study reported that they would prefer materials that rely less on text and more on graphics, focus on specific behavior change recommendations, include simple advice to make healthier food choices, and list foods to eat more of or to avoid. In a prior analysis from this study, we found that many CHC clinicians reported that they have begun to develop their own tools, handouts and resources to give patients during clinical encounters [22]. Future avenues for research may include developing and evaluating materials for clinicians to use during weight counseling encounters that are better suited to meeting the needs of CHC patients.

CHC clinicians also reported that their patients seem to lack interest in and motivation to lose weight. This challenges findings from a recent national survey, which found that the majority (60.1 %) of overweight and obese CHC patients reported that they had tried to lose weight in the past year, most frequently through dietary modification and exercise or by dietary modification alone [9]. The demographic and socioeconomic characteristics of the patient population at the CHCs included in this analysis are similar to CHC patients nationwide, suggesting that differences in these characteristics do not explain these discrepancies in patient self-report and provider perceptions. For example, HRSA estimates that in 2013, 62.9 % of national CHC patients were racial or ethnic minorities, 92.8 % were at or below 200 % of the federal poverty level, and 34.9 % were uninsured [25]. By comparison, at the CHCs included in this study, 52.3–76.5 % of patients were racial or ethnic minorities, 74.1–99.0 % were at or below 200 % of the federal poverty level, and 23.3–31.0 % were uninsured [25]. Future research should explore reasons for this discrepancy in provider and patient perceptions and the extent to which CHC clinicians are aware of and use motivational interviewing or the five As technique, which have been tested as strategies to assist clinicians with providing obesity-related counseling during brief clinical encounters [26, 27]. In light of findings from previous studies that clinicians report lack of training as a barrier to obesity counseling [28], future research should explore the extent to which trainings on these techniques could better equip clinicians to counsel patients they perceive to be unmotivated.

Clinicians also perceived higher-order interpersonal and community determinants as barriers to healthy weight maintenance among their patients. Interpersonal barriers identified by clinicians included non-health-promoting social norms, such as positive associations with larger body sizes, negative associations with smaller body sizes, and the belief that obesity is normative or inevitable among patient communities. Community-level barriers identified by clinicians included the limited availability of healthy food options and aspects of Southern food culture. Interestingly, clinicians did not discuss a number of community and organizational-level determinants of obesity that have been identified by prior research in southwest Georgia [29–32]. For example, clinicians in this study tended to describe barriers to healthy eating and did not focus on aspects of the built environment that impede physical activity, including the lack of sidewalks, public parks, and recreation areas. Additionally, clinicians did not discuss non-health-promoting aspects of organizational environments within churches, schools, and workplaces as important contextual influences.

These findings suggest that clinicians perceive their patients to face complex individual and environmental determinants of obesity, which cannot be effectively addressed during brief clinical encounters. Although not directly assessed as part of this study, it is possible that these perceived patient barriers, in combination with physician and clinic-level barriers identified by previous studies [12, 17–22], may partly explain why the provision of weight counseling in CHCs remains low. In 2014, HRSA estimated that 56.1 % of adult CHC patients received weight screening and follow-up [7], and according to a survey of overweight and obese CHC patients, less than half (45.2 %) reported that a physician told them they were overweight [16]. It is possible that clinicians prioritize other health-related concerns during their brief clinical encounters with patients when they perceive that their patients are unable to access or afford healthy foods, will have difficulty understanding complex nutrition education materials, have limited social support for weight loss within their social networks or communities, or have limited motivation to act on weight loss advice. This may partly explain the limited provision of weight counseling in CHCs.

These findings also have implications for intervention development. Consistent with ecological theories of health promotion [24, 33], these findings suggest that CHC clinicians and patients may benefit from partnering with public health researchers and practitioners to develop, adapt, and implement evidence-based multilevel interventions to address individual, interpersonal, and community-level determinants of obesity. This recommendation is consistent with growing interest in forming community-clinic partnerships to address obesity and chronic disease prevention [12, 34, 35]. Such partnerships may enable public health practitioners to reach populations that may benefit from weight management interventions, and may enable clinicians to more effectively address weight with their patients by providing them with needed weight management referral resources [22].

This study had several limitations, which must be acknowledged in order to correctly interpret the results. We used qualitative research methods to document the perceptions and experiences of primary care clinicians practicing at CHCs in rural, southwest Georgia. These results are not intended to represent the views of all primary care clinicians at CHCs, nor are they intended to generalize to other clinical settings or geographic areas. Additionally, these results reflect clinicians’ perspectives on their patients’ experiences and may differ from what patients identify as the major barriers they face in successful weight loss.

## Conclusions

Despite these limitations, these results provide insight into the barriers that CHC clinicians perceive their patients face in maintaining a healthy weight. These results may contribute to an increased understanding of why the provision of weight counseling remains low in CHCs and suggest opportunities for improving obesity control practices in this high priority clinical setting.

## Abbreviations

BMI: Body mass index
CHCs: Community Health Centers
HRSA: Health Resources and Services Administration
US: United States

## References

[1] Ogden CL, Carroll MD, Kit BK, Flegal KM (2014). Prevalence of childhood and adult obesity in the United States, 2011–2012. JAMA.

[2] Guh DP, Zhang W, Bansback N, Amarsi Z, Birmingham CL, Anis AH (2009). The incidence of co-morbidities related to obesity and overweight: a systematic review and meta-analysis. BMC Public Health.

[3] Wang YC, McPherson K, Marsh T, Gortmaker SL, Brown M (2011). Health and economic burden of the projected obesity trends in the USA and the UK. Lancet.

[4] Wang Y, Beydoun MA (2007). The obesity epidemic in the United States - Gender, age, socioeconomic, Racial/Ethnic, and geographic characteristics: A systematic review and meta-regression analysis. Epidemiol Rev.

[5] Befort CA, Nazir N, Perri MG (2012). Prevalence of obesity among adults from rural and urban areas of the United States: findings from NHANES (2005–2008). J Rural Health.

[6] Adashi EY, Geiger HJ, Fine MD (2010). Health care reform and primary care--the growing importance of the community health center. N Engl J Med.

[7] Health Resources and Services Administration. Health Center Data 2014. [cited August 2016]. Available from: http://bphc.hrsa.gov/uds/datacenter.aspx.

[8] Shin P, Alvarez C, Sharac J, Rosenbaum S, Van Vleet A, Paradise J, et al. A Profile of Community Health Center Patients: Implications for Policy. 2013 [cited August 2015]. Available from: https://kaiserfamilyfoundation.files.wordpress.com/2013/12/8536-profile-of-chc-patients.pdf.

[9] Lebrun LA, Chowdhury J, Sripipatana A, Nair S, Tomoyasu N, Ngo-Metzger Q (2013). Overweight/obesity and weight-related treatment among patients in U.S. federally supported health centers. Obes Res Clin Pract.

[10] Linde S, Coulouris N, Heppner E. Advancing obesity prevention: emerging models and best practices. J Health Care Poor Underserved. 2013;24(2 Suppl):viii-xii 10.1353/hpu.2013.0105 23727976

[11] Moyer VA, Force UPST. Screening for and Management of Obesity in Adults: US Preventive Services Task Force Recommendation Statement. Ann Intern Med. 2012;157(5):373-U126 10.7326/0003-4819-157-5-201209040-00475 22733087

[12] Allen CL, Harris JR, Hannon PA, Parrish AT, Hammerback K, Craft J (2014). Opportunities for Improving Cancer Prevention at Federally Qualified Health Centers. J Cancer Educ.

[13] Health Resources and Services Administration Bureau of Primary Health Care. Clinical and Financial Performance Measures. [cited July 2014]. Available from: http://bphc.hrsa.gov/policiesregulations/performancemeasures/.

[14] Kraschnewski JL, Sciamanna CN, Stuckey HL, Chuang CH, Lehman EB, Hwang KO (2013). A silent response to the obesity epidemic: decline in US physician weight counseling. Med Care.

[15] Bleich SN, Pickett-Blakely O, Cooper LA (2011). Physician practice patterns of obesity diagnosis and weight-related counseling. Patient Educ Couns.

[16] Post RE, Mainous AG, Gregorie SH, Knoll ME, Diaz VA, Saxena SK (2011). The influence of physician acknowledgment of patients’ weight status on patient perceptions of overweight and obesity in the United States. Arch Intern Med.

[17] Leverence RR, Williams RL, Sussman A, Crabtree BF, Clinicians RN (2007). Obesity counseling and guidelines in primary care: a qualitative study. Am J Prev Med.

[18] Alexander SC, Ostbye T, Pollak KI, Gradison M, Bastian LA, Brouwer RJN (2007). Physicians’ beliefs about discussing obesity: Results from focus groups. Am J Health Promot.

[19] Sussman AL, Williams RL, Leverence R, Gloyd PW, Crabtree BF (2006). The art and complexity of primary care clinicians’ preventive counseling decisions: Obesity as a case study. Ann Fam Med.

[20] Forman-Hoffman V, Little A, Wahls T (2006). Barriers to obesity management: a pilot study of primary care clinicians. BMC Fam Pract.

[21] Huang J, Yu H, Marin E, Brock S, Carden D, Davis T (2004). Physicians’ weight loss counseling in two public hospital primary care clinics. Acad Med.

[22] Schauer GL, Woodruff RC, Hotz J, Kegler MC (2014). A qualitative inquiry about weight counseling practices in community health centers. Patient Educ Couns.

[23] Hennink M, Hutter I, Bailey A. Qualitative research methods. Thousand Oaks: Sage Publications Inc.; 2011.

[24] McLeroy KR, Bibeau D, Steckler A, Glanz K (1988). An ecological perspective on health promotion programs. Health Educ Q.

[25] Health Resources and Services Administration. Health Center Program Grantee Profiles 2013. [cited August 2016]. Available from: http://bphc.hrsa.gov/uds/datacenter.aspx?q=d&year=2015&state=GA#glist.

[26] Vallis M, Piccinini-Vallis H, Sharma AM, Freedhoff Y (2013). Modified 5 As Minimal intervention for obesity counseling in primary care. Can Fam Physician.

[27] Armstrong M, Mottershead T, Ronksley P, Sigal R, Campbell T, Hemmelgarn B (2011). Motivational interviewing to improve weight loss in overweight and/or obese patients: a systematic review and meta‐analysis of randomized controlled trials. Obes Rev.

[28] Jay M, Kalet A, Ark T, McMacken M, Messito MJ, Richter R (2009). Physicians’ attitudes about obesity and their associations with competency and specialty: a cross-sectional study. BMC Health Serv Res.

[29] Escoffery C, Kegler MC, Alcantara I, Wilson M, Glanz K (2011). A qualitative examination of the role of small, rural worksites in obesity prevention. Prev Chronic Dis.

[30] Kegler MC, Swan DW, Alcantara I, Wrensford L, Glanz K (2012). Environmental influences on physical activity in rural adults: The relative contributions of home, church, and work settings. J Phys Act Health.

[31] Kegler MC, Escoffery C, Alcantara I, Ballard D, Glanz K (2008). A qualitative examination of home and neighborhood environments for obesity prevention in rural adults. Int J Behav Nutr Phys Act.

[32] Williams RM, Glanz K, Kegler MC, Davis E (2012). A study of rural church health promotion environments: leaders’ and members’ perspectives. J Relig Health.

[33] Frieden TR (2010). A framework for public health action: the health impact pyramid. Am J Public Health.

[34] Sequist TD, Taveras EM (2014). Clinic-community linkages for high-value care. N Engl J Med.

[35] Freedman DA, Pena-Purcell N, Friedman DB, Ory M, Flocke S, Barni MT (2014). Extending cancer prevention to improve fruit and vegetable consumption. J Cancer Educ.

